# Neonatal blood pressure by birth weight, gestational age, and postnatal age: a systematic review

**DOI:** 10.1186/s40748-024-00180-w

**Published:** 2024-05-01

**Authors:** Rhys Dore, Katy Barnes, Stephen Bremner, Hiroko Ishii Iwami, Dina Apele-Freimane, Beau Batton, Eugene Dempsey, Ebru Ergenekon, Agnes Klein, Luana Pesco-Koplowitz, Janis M. Dionne, Heike Rabe

**Affiliations:** 1 Royal London Hospital, Barts Health NHS Trust, London, UK; 2 Department of Neonatology, University Hospitals Sussex, Brighton, UK; 3 Brighton and Sussex Medical School, University of Sussex, Brighton, UK Eastern Road, BN2 5BE; 4 Osaka City General Hospital, Osaka, Japan; 5 Pauls Stradins Clinical University Hospital, Riga, Latvia; 6 Southern Illinois University School of Medicine, Springfield, IL USA; 7 University College Cork, Cork, Ireland; 8 Gazi University, Ankara, Turkey; 9 Health Canada, Ottawa, Canada; 10 DUCK FLATS Pharma LLC, Flemington, New Jersey USA; 11 British Columbia Children´S Hospital, Vancouver, Canada

**Keywords:** Neonatology, Blood pressure, Hypertension, Hypotension, Prematurity, Birth weight

## Abstract

**Background:**

Blood pressure is a vital hemodynamic marker during the neonatal period. However, normative values are often derived from small observational studies. Understanding the normative range would help to identify ideal thresholds for intervention to treat hypotension or hypertension. Therefore, the aim of this study was to assess observed blood pressure values in neonates who have not received any blood-pressure modifying treatments from birth to three months postnatal age and whether these vary according to birth weight, gestational age and postnatal age.

**Methods:**

This was a systematic review. A literature search was conducted in MEDLINE, PubMed, Embase, Cochrane Library, and CINAHL from 1946 to 2017 on blood pressure in neonates from birth to 3 months of age (PROSPERO ID CRD42018092886). Unpublished data were included where appropriate.

**Results:**

Of 3,587 non-duplicate publications identified, 30 were included (one unpublished study). Twelve studies contained data grouped by birth weight, while 23 contained data grouped by gestational age. Study and clinical heterogeneity precluded meta-analyses thus results are presented by subgroup. A consistent blood pressure rise was associated with increasing birth weight, gestational age, and postnatal age. In addition, blood pressure seemed to rise more rapidly in the most preterm and low birth weight neonates.

**Conclusion:**

Despite blood pressure increasing with birth weight, gestational age, and postnatal age, there was marked blood pressure variability observed throughout. To better define hypotension and hypertension, future studies should develop consistent approaches for factors related to blood pressure variability, including the method and timing of measurement as well as statistical control of relevant patient characteristics.

**Supplementary Information:**

The online version contains supplementary material available at 10.1186/s40748-024-00180-w.

## Background

Blood pressure (BP) is one of the vital parameters measured after birth to assess how a neonate is adjusting to extrauterine life. Normative data for term and late preterm neonates has generally been derived from small observational studies in which BP values were obtained using a variety of methods over a relatively narrow time frame [1, 2]. A previous assessment found that the number of patients included in these studies ranged from as little as 16 to 608, whilst our review has found studies ranging from 4 to 3079 [3]. For the last three decades, “normal” BP values for extremely preterm neonates have commonly been defined as a mean arterial BP value in millimeters of mercury (mmHg) that is numerically above the neonate’s gestational age (GA) at birth (in weeks) – even though there is little evidence this definition is indicative of circulatory compromise [4]. There are no well-defined thresholds for clinically relevant low BP (hypotension) for which any therapeutic intervention has been shown to consistently improve outcome in neonates. This may also be at least partly because low BP may be a sub-optimal marker for hemodynamic assessment and end-organ perfusion [5, 6]. In addition, neonatal hypertension is defined by elevated BP percentiles extrapolated from definitions in older children and not from established associations with any cardiovascular or patient outcomes in the neonatal period or further [7]. While other measures of cardiovascular assessment in neonates are being investigated and, in some instances, utilized to direct care, BP (along with heart rate) remain the most frequently measured assessment of the cardiovascular system in the neonatal intensive care unit (NICU) [1, 2].

Challenges exist regarding how to best measure BP in the neonate accurately and reliably [2]. Intra-arterial measurement is considered the “gold-standard”, but is invasive and not practical for many neonates. Therefore, less reliable methods are often utilized – most commonly the oscillatory method. With oscillometric devices, variability can occur based on integrated algorithms, cuff placement, state (i.e., sleeping, crying) during measurement and whether one-off or repeated measurements are taken. In addition to these challenges, preterm neonates and those admitted to the NICU are an inherently abnormal population with typical “observed” values that may deviate from “normal” readings within the healthy term population. Notably, there are established discrepancies between the previously established normative ranges within the neonatal period [3]. This is due to the prevalence of multiple small heterogeneous studies, whilst larger reviews have more commonly focused on longer term associations between blood pressure, neonatal parameters, and disease rather than determining normative values [8–11].

The purpose of this study was to develop population based normative BP data derived from published original data. From these calculations, high and low BP values for various populations of neonates can be identified. These values could then be used to identify potential thresholds for therapeutic interventions directed to correcting hypertension or hypotension in both term and preterm neonates in future clinical drug trials. This systematic review examined the question: What are the ranges of BP values observed in neonates who have not received any blood-pressure modifying treatments based on the following: birth weight (BW), gestational age (GA), postnatal age (PNA)?

## Methods

A literature search was conducted for studies published from January 1946 to December 2017 that contained data regarding blood pressure measurements for neonates between birth and 3 months PNA that were not hemodynamically compromised or requiring BP modifying agents. Unpublished data from research undertaken by consortium members was also considered for inclusion. All studies were reviewed by at least two independent reviewers and all co-authors were involved in study review and data extraction. Planned analysis of extracted data was descriptive as formal statistical comparisons were not possible. Summary statistics were extracted for systolic blood pressure (SBP), diastolic blood pressure (DBP) and mean arterial pressure (MAP). In addition to this, data regarding BW, GA, and PNA were collected and grouped for presentation. Additional files have been provided with a detailed methodology (Additional File 1) and the PRISMA 2020 Standard (Additional File 2). The protocol for this review is registered on the PROSPERO website with ID CRD42018092886.

## Results

**Fig. 1:**
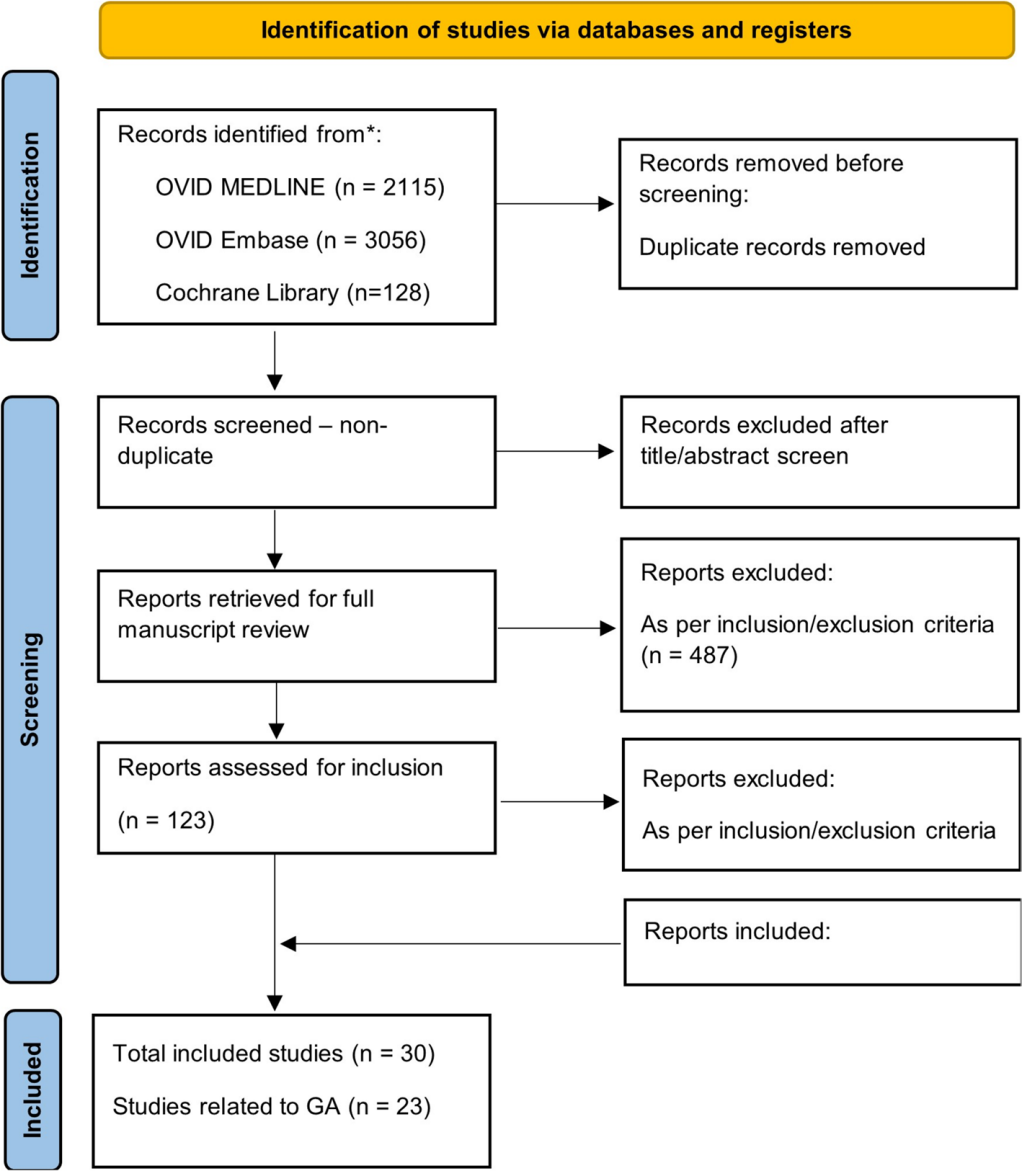
PRISMA Flowchart

The systematic search identified 5299 reports. After removing duplicates, 3587 titles and abstracts were reviewed by 11 members of the working group, with 623 articles extracted for in-depth review. Of these, 123 were assessed and found to specifically apply to the review question that is the focus of the current manuscript. In total, 29 published studies and 1 unpublished study were included. Twenty-three studies contained extractable data grouped by GA [1, 12–33]. Twelve studies contained extractable data grouped by BW [1, 16, 25, 26, 32, 34–40]. The selection process is illustrated in Fig. 1 (PRISMA flow chart). Generally, risk of bias was low with minimal concerns reported in the qualitative assessment, particularly for descriptive BP data – therefore all included studies have been presented. A brief description of each included study is provided in Additional File 3, with extracted data presented in Additional File 4.

## Blood pressure and gestational age at birth

### Term neonates

#### Summary results

**Fig. 2:**
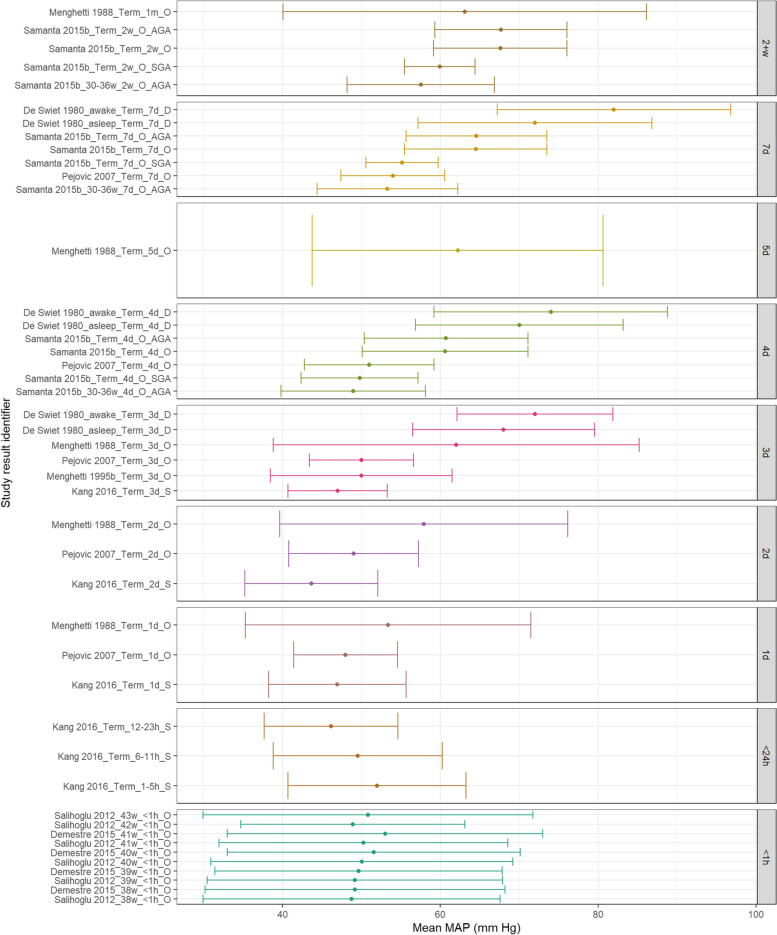
MAP in term babies by postnatal age. Mean BP (mmHg) and 90% reference ranges (1.64SD) for term and post-term infants. Results reported from < 1 h to 1 month postnatal age. Results are sorted in ascending order of PNA. The labels on the vertical axes give the following information: author & year_(subgroup, if applicable)_GA_PNA_method where method is method of BP measurement used (D = doppler, I = intra-arterial, O = oscillometric, C = noninvasive photoplethysmographic cuff, S = Sphygmomanometer)

**Fig. 3:**
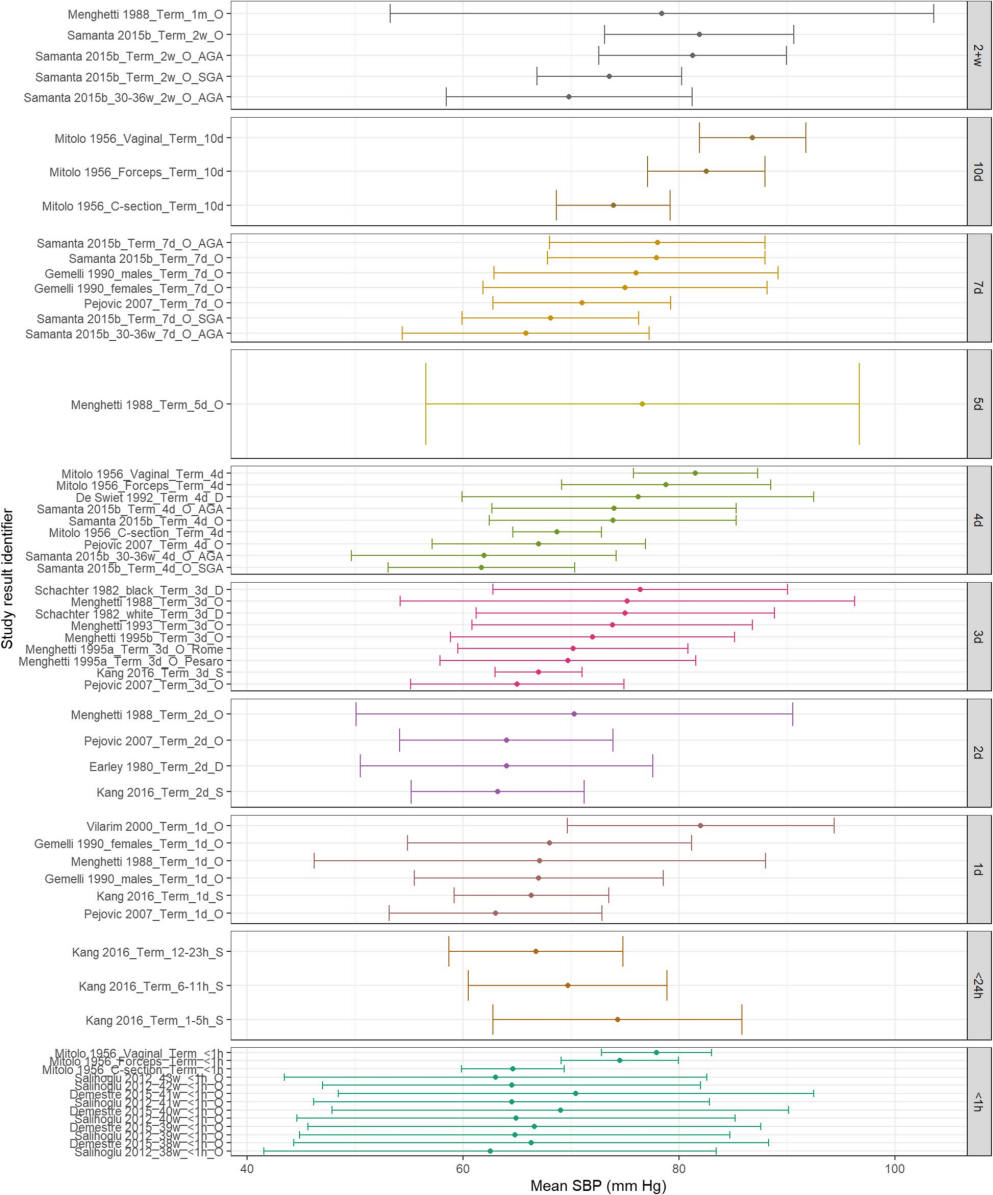
SBP in term babies by postnatal age. Mean BP (mmHg) and 90% reference ranges (1.64SD) for term and post-term infants. Results reported from < 1 h to 1 month postnatal age. Results are sorted in ascending order of PNA. The labels on the vertical axes give the following information: author & year_(subgroup, if applicable)_GA_PNA_method where method is method of BP measurement used (D = doppler, I = intra-arterial, O = oscillometric, C = noninvasive photoplethysmographic cuff, S = Sphygmomanometer)

**Fig. 4:**
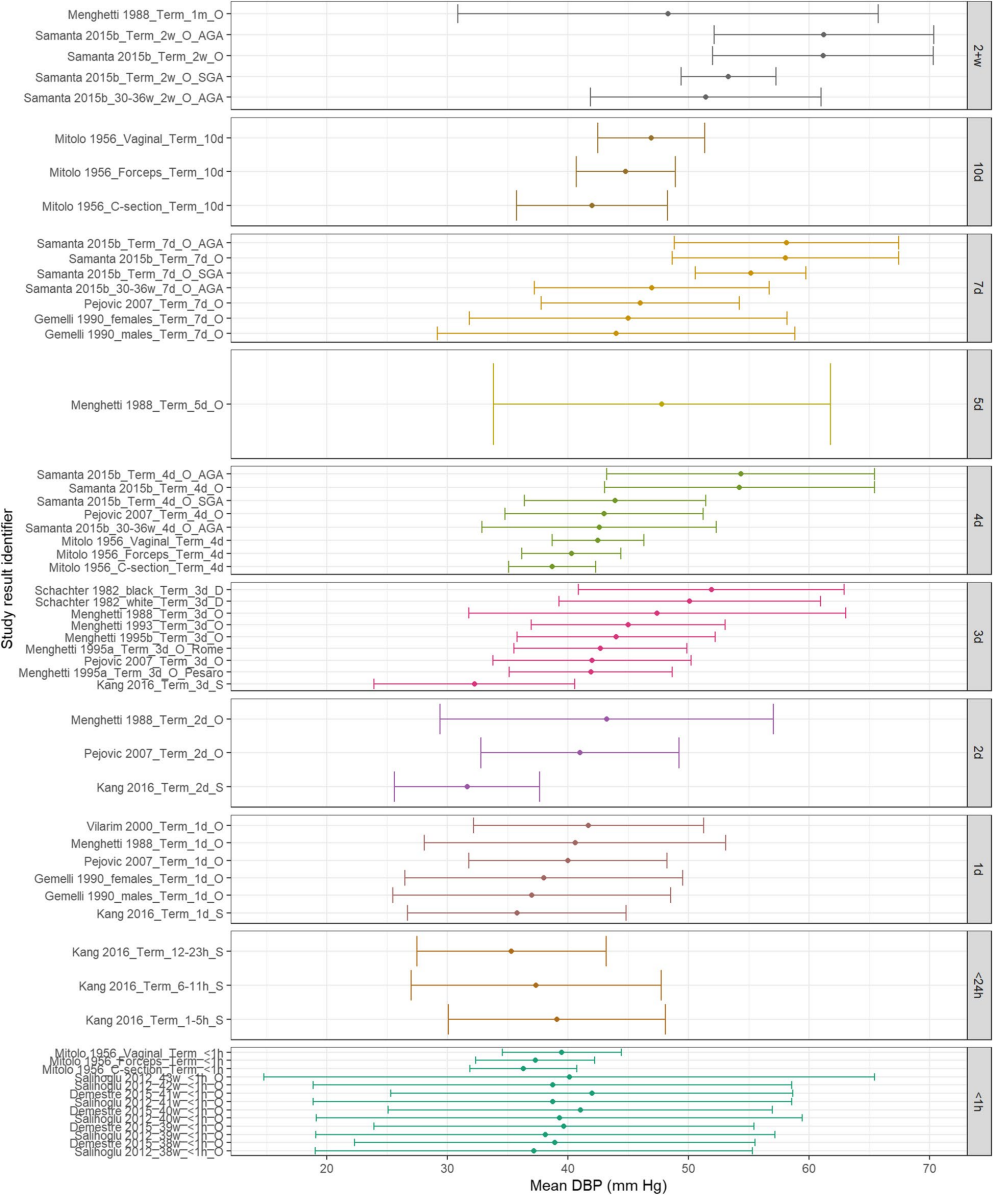
DBP in term babies by postnatal age. Mean BP (mmHg) and 90% reference ranges (1.64SD) for term and post-term infants. Results reported from < 1 h to 1 month postnatal age. Results are sorted in ascending order of PNA. The labels on the vertical axes give the following information: author & year_(subgroup, if applicable)_GA_PNA_method where method is method of BP measurement used (D = doppler, I = intra-arterial, O = oscillometric, C = noninvasive photoplethysmographic cuff, S = Sphygmomanometer)

Figure 2 shows that MAP appears similar at < 1 h of age with mean values between 45-55 mmHg. Despite some variability, this appears to rise demonstrably over the first 7-days of life but further increases were not noted as neonates approached 1 month of life. Figure 3 demonstrates that SBP appears to increase with increasing PNA for term neonates. Figure 4 demonstrates that DBP appears similar within 1 day of life but then demonstrated a gradual increase with increasing PNA.

#### Study descriptions

Eighteen studies were identified as investigating BP in term neonates including a total of 14,557 neonates. Of these, five used Doppler methods of BP measurement with three Doppler studies examining BP within the first 72 h of life. Schachter et al. reported 392 term neonates on postnatal day 3 [29]. Earley et al. monitored SBP in 99 infants between postnatal day two and six weeks of age [17]. They demonstrated a rise in SBP of 14 mmHg in the first two weeks with a further rise of 9 mmHg by six weeks of age. Kang et al. used doppler ultrasound to measure brachial artery pressures in 76 term neonates with patent ductus arteriosus in the first 72 h of life [20]. De Swiet et al. published two studies relating to BP in term neonates over a longer time period [14, 15]. The 1980 study repeatedly measured systolic BP by Doppler ultrasound in 391 sleeping and awake term neonates at 4—6 days and then again at 5—7 weeks. In sleeping neonates, a rise in BP was noted over this time frame from 70.7 mmHg ± 0.3 mm Hg to 89.7 mmHg ± 0.9 mmHg. A similar rise was also seen in neonates who were awake. Observed BP values during both time frames seemed to be related as neonates with higher values at 4—6 days also had higher values at 5—7 weeks. De Swiet et al.’s later study reported on both doppler and sphygmomanometer BP measurements for 1740 term neonates (both asleep and awake) between four days and one year of age. Extractable data were identified for those in the first week with an increasing trend over this time.

Thirteen studies used oscillometric measurements to examine BP. Eight of these studies only contained extractable data within the first 72 h of life. Salihoglu et al. measured BP in the delivery room for 982 neonates [26]. Demestre et al. measured BP between 30 and 60 min after birth for 4496 neonates [16]. Vilarim and Alves measured BP in 634 neonates between 12 and 36 h of age [30]. Satoh et al. measured BP in an arm and leg for 3088 neonates on day 3 [28]. Menghetti et al. published four studies of BP measurements in term neonates: 1) 160 neonates with BP measured at one month, 2) 105 neonates with BP measured within 72 h of birth, 3) 150 neonates with BP measured over the first 72 h after birth, 4) 16 neonates who were less than four days old [21–24]. Five studies used oscillometric measurements and contained extractable data beyond the first 72 h of life. Samanta et al. reported BP values for 1427 term infants on days four, seven, and 2 weeks [27, 33]. BP values were significantly higher in this group compared to the preterm cohort and increased with PNA. Pejovic et al. recorded BP values for 81 term neonates through the first 30 days after birth and also reported an increase in BP with advancing PNA. [1] Gemelli et al. measured BP in 514 neonates between birth and 12 months [18]. They noted a statistically significant increase in diastolic and systolic BP from birth to six months of age (mean differences of 29 mmHg and 21 mmHg respectively), with minimal differences between BP at 6 months and 1 year. Mitolo and Grassi provided measurements for 46 neonates within the first 10 days of life [25].

## Preterm neonates

### Summary results

**Fig. 5:**
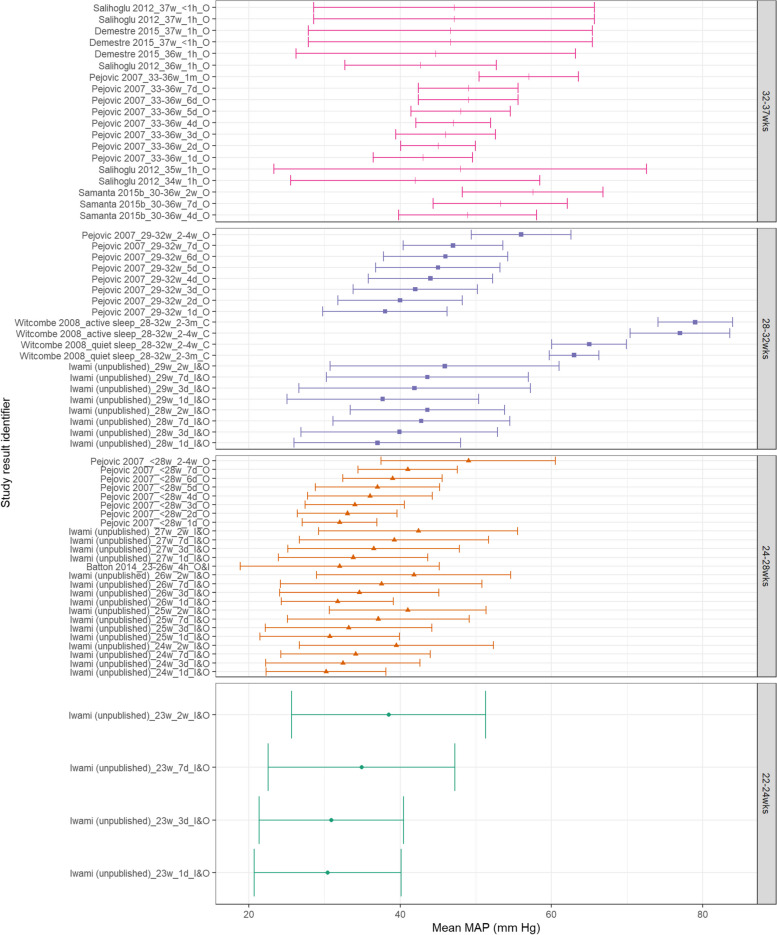
MAP in preterm babies by postnatal age. Mean BP (mmHg) and 90% reference ranges (1.64SD) for preterm infants with GA 22^0^–23^6^ weeks, 24^0^–27^6^ weeks, 28^0^–31^6^ weeks, and 32^0^–36.^6^ weeks. Results report from 1 h to 2–3 months postnatal age, sorted in descending order of prematurity, and within those by PNA. The labels on the vertical axis give the following information: author & year_(subgroup, if applicable)_GA_PNA_method where method is method of BP measurement used (D = Doppler, I = intra-arterial, O = oscillometric, C = noninvasive photoplethysmographic cuff, S = Sphygmomanometer)

**Fig. 6:**
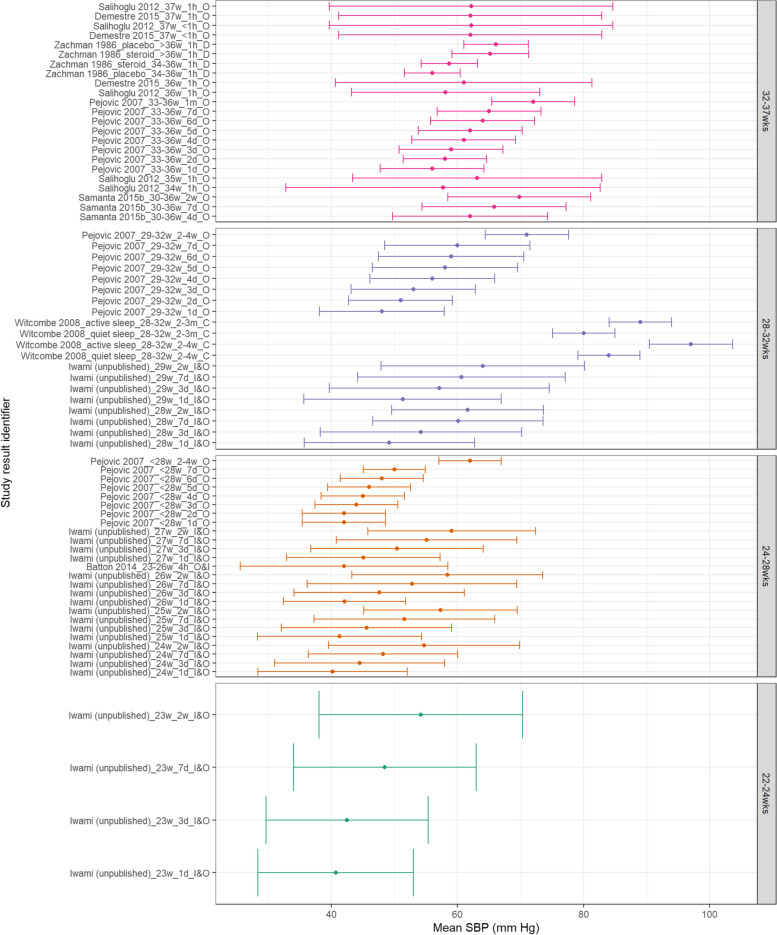
SBP in preterm babies by postnatal age. Mean BP (mmHg) and 90% reference ranges (1.64SD) for preterm infants with GA 22^0^–23^6^ weeks, 24^0^–27^6^ weeks, 28^0^–31^6^ weeks, and 32^0^–36.^6^ weeks. Results report from 1 h to 2–3 months postnatal age, sorted in descending order of prematurity, and within those by PNA. The labels on the vertical axis give the following information: author & year_(subgroup, if applicable)_GA_PNA_method where method is method of BP measurement used (D = Doppler, I = intra-arterial, O = oscillometric, C = noninvasive photoplethysmographic cuff, S = Sphygmomanometer)

**Fig. 7:**
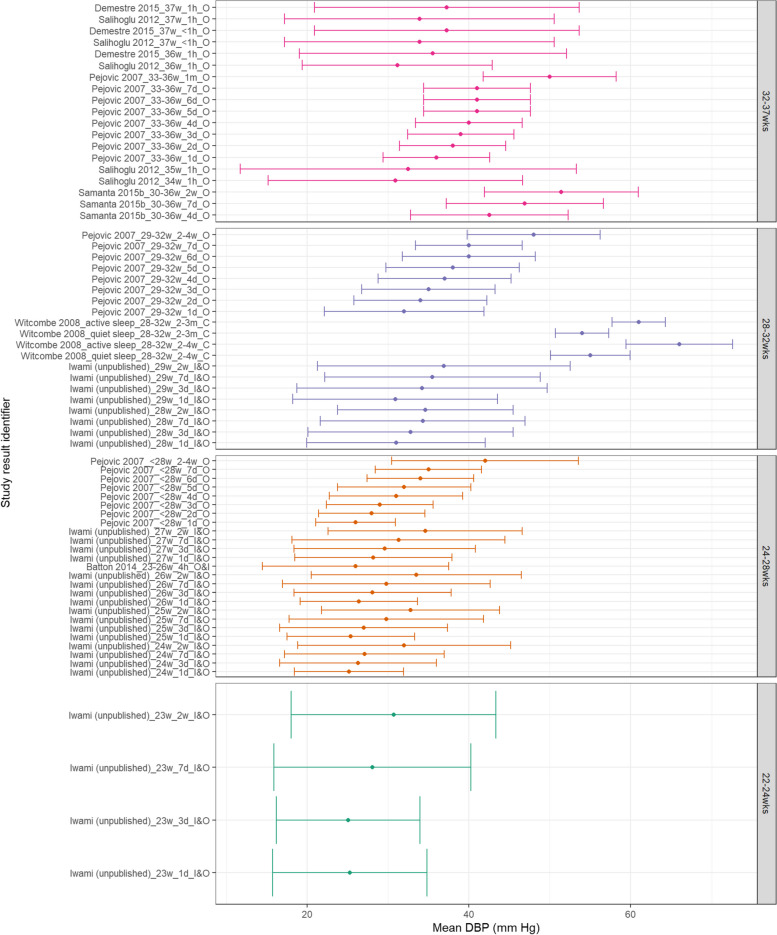
DBP in preterm babies by postnatal age. Mean BP (mmHg) and 90% reference ranges (1.64SD) for preterm infants with GA 22^0^-23^6^ weeks, 24^0^-27^6^ weeks, 28^0^-31^6^ weeks, and 32^0^-36.^6^ weeks. Results report from 1 h to 2-3 months postnatal age, sorted in descending order of prematurity, and within those by PNA. The labels on the vertical axis give the following information: author & year_(subgroup, if applicable)_GA_PNA_method where method is method of BP measurement used (D = Doppler, I = intra-arterial, O = oscillometric, C = noninvasive photoplethysmographic cuff, S = Sphygmomanometer)

Within Fig. 5, MAP appears to consistently increase with increasing PNA and generally with increasing GA. SBP also appears to increase with increasing GA and PNA as shown in Fig. 6. Within both Figs. 5 and 6, data from the 28^0/7^–31^6/7^ week categories at postnatal age > 2 weeks is higher than both more and less preterm categories. This is notably due to Witcombe et al.’s study demonstrating higher BP readings at 2–4 weeks PNA and 2–3 months PNA – this is especially true for measurements of neonates within an active state. Similarly, Fig. 7 shows that DBP appears to increase with increasing PNA. DBP also appears to increase with increasing GA, although this difference is less distinct between the 28^0/7^–31^6/7^ weeks and 32^0/7^–36^6/7^ weeks categories.

**Fig. 8:**
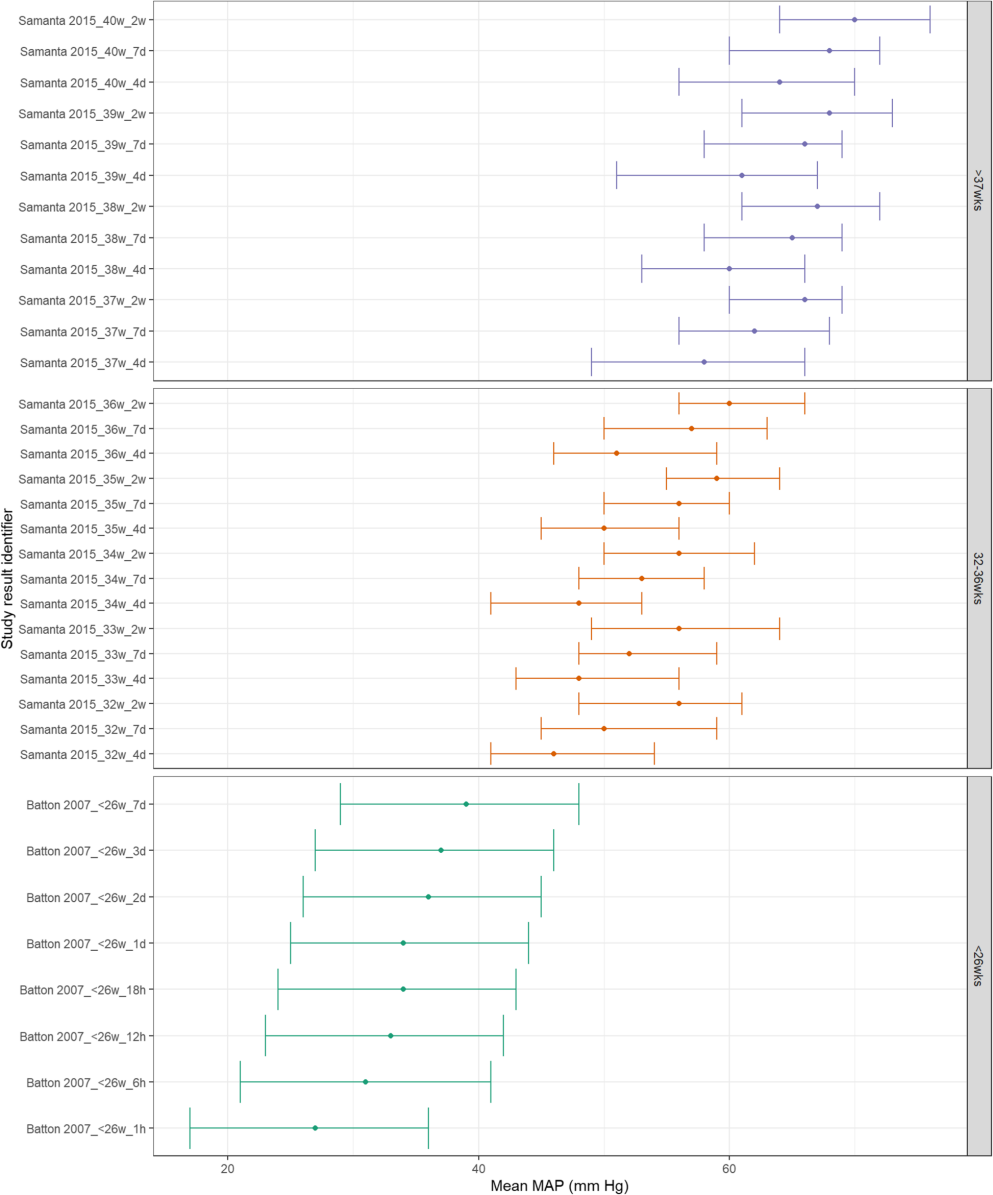
MAP in term and preterm infants by postnatal age (medians). Median BP (mmHg) and 10th-90th centile range for preterm and term infants with GA < 26^0^ weeks, 32^0^ – 36^6^ weeks, > 37^0^ weeks. Results reported from 1 h to 2 weeks postnatal age and sorted in ascending order of prematurity and within those by PNA. The labels on the vertical axis give the following information: author & year_(subgroup, if applicable)_GA_PNA

**Fig. 9:**
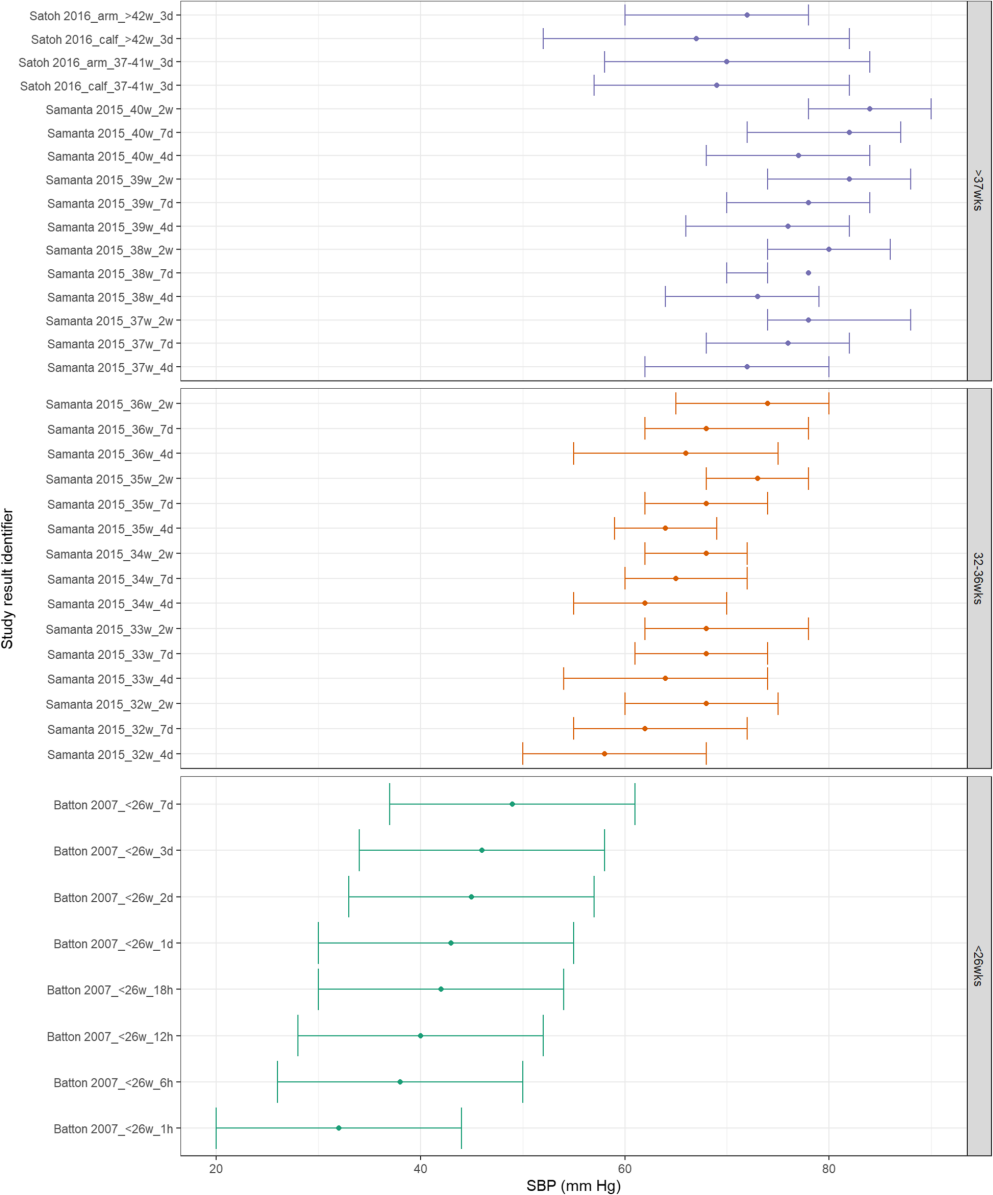
SBP in term and preterm infants by postnatal age (medians). Median BP (mmHg) and 10th-90th centile range for preterm and term infants with GA < 26^0^ weeks, 32^0^ – 36^6^ weeks, > 37^0^ weeks. Results reported from 1 h to 2 weeks postnatal age and sorted in ascending order of prematurity and within those by PNA. The labels on the vertical axis give the following information: author & year_(subgroup, if applicable)_GA_PNA

**Fig. 10:**
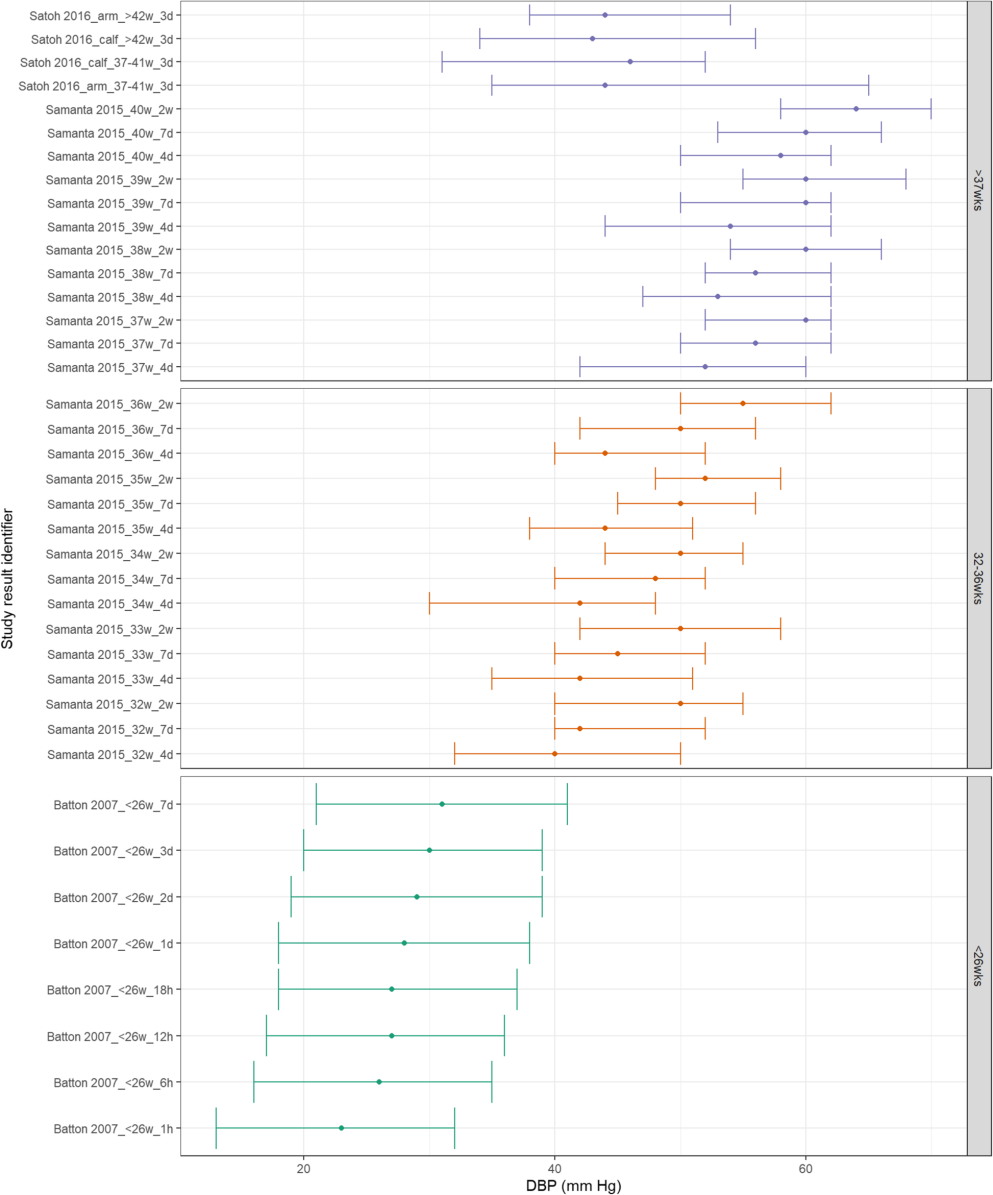
C: DBP in term and preterm infants by postnatal age (medians). Median BP (mmHg) and 10th-90th centile range for preterm and term infants with GA < 26^0^ weeks, 32^0^ – 36^6^ weeks, > 37^0^ weeks. Results reported from 1 h to 2 weeks postnatal age and sorted in ascending order of prematurity and within those by PNA. The labels on the vertical axis give the following information: author & year_(subgroup, if applicable)_GA_PNA

Studies reporting medians rather than means were collated for visual presentation. Figure 8 shows MAP appears to increase consistently with increasing postnatal age and with increasing GA. These differences between GA subgroups are still evident at both 7 days and 2 weeks postnatal age. Similarly, both SBP and DBP demonstrate a similar increase with increasing GA and postnatal age as shown in Figs. 9 and  10.

### Study descriptions

Eight studies were identified investigating BP in preterm neonates plus an additional unpublished data set that was also available for analysis resulting in a total of 1334 patients. Five studies included data for late preterm neonates. Demestre et al. reported oscillometric BP values at 30 – 60 min of age in 198 neonates born at 36^0/7^ to 36^6/7^ weeks GA, with increasing BP with GA [16]. Salihoglu et al. reported delivery room BP values in 46 neonates born at 34^0/7^ to 36^6/7^ weeks GA [26]. Samanta et al. published BP data on postnatal days 4, 7, and 1 days from 190 preterm neonates born at 32^0/7^ to 36^6/7^ weeks GA [27, 33]. Centile charts from this cohort demonstrated increased BP values with both increasing GA and PNA. Pejovic et al. examined oscillometric BP measurements over the first month after birth in 292 preterm neonates grouped into three cohorts, including 81 born 33^0/7^ to 36^6/7^ weeks GA [1]. Both systolic and diastolic BP increased over the month with a higher rate of rise seen in preterm neonates compared to the ≥ 37^0/7^ group. Zachman et al. reported BP values within the first hour of life for a cohort of 225 neonates born from 30^0/7^ weeks GA to term subdivided based on receipt of antenatal corticosteroids [32]. Although they also reported higher BP values with increasing GA at birth, BP values were not significantly different based on antenatal corticosteroid exposure.

Two published studies included data for very to moderately preterm neonates born at 28^0/7^ to 32^6/7^ weeks GA. Witcombe et al. examined BP using a photoplethysmographic cuff for 25 preterm neonates at 2—4 weeks and 2 -3 months of age: BP pressure remained static over this time period, but increased when neonates were in an active versus quiet state [31]. Pejovic et al. studied 146 neonates born at 29^0/7^ to 32^6/7^ weeks GA, with further data collected for 62 neonates < 29^0/7^ weeks GA. Unpublished data were available from Iwami (unpublished data from the PLASE database described by Toyoshima et al.) for 62 neonates between 28^0/7^ to 29^6/7^ weeks GA over the first 2 weeks of life [19]. BP values were obtained using both invasive and oscillometric measurements. An increase in BP was seen with increasing GA at birth and advancing PNA.

Data for the extremely preterm group had been obtained using a mixture of invasive arterial monitoring and oscillometric measurements. Iwami’s unpublished data reported a further 98 infants born 23^0/7^ to 27^6/7^ [19]. Batton et al. published BP data over the first postnatal week for 86 neonates born at 23^0/7^ to 25^6/7^ weeks GA who did not receive treatment for hypotension [12, 13]. They demonstrated a rise in mean arterial BP by 0.3 mmHg/hour in the first 24 h and 0.1 mmHg/hour between 24 and 48 h with minimal changes noted between 48 h and 7 days. A second study by Batton presented hourly BP data for the first 24 h after birth from 164 neonates born at 23^0/7^ to 26^6/7^ weeks who also did not receive anti-hypotensive therapy. Mean BPs and rate of rise between 4 and 24 h were presented.

## Blood pressure and birthweight

### Summary results

**Fig. 11:**
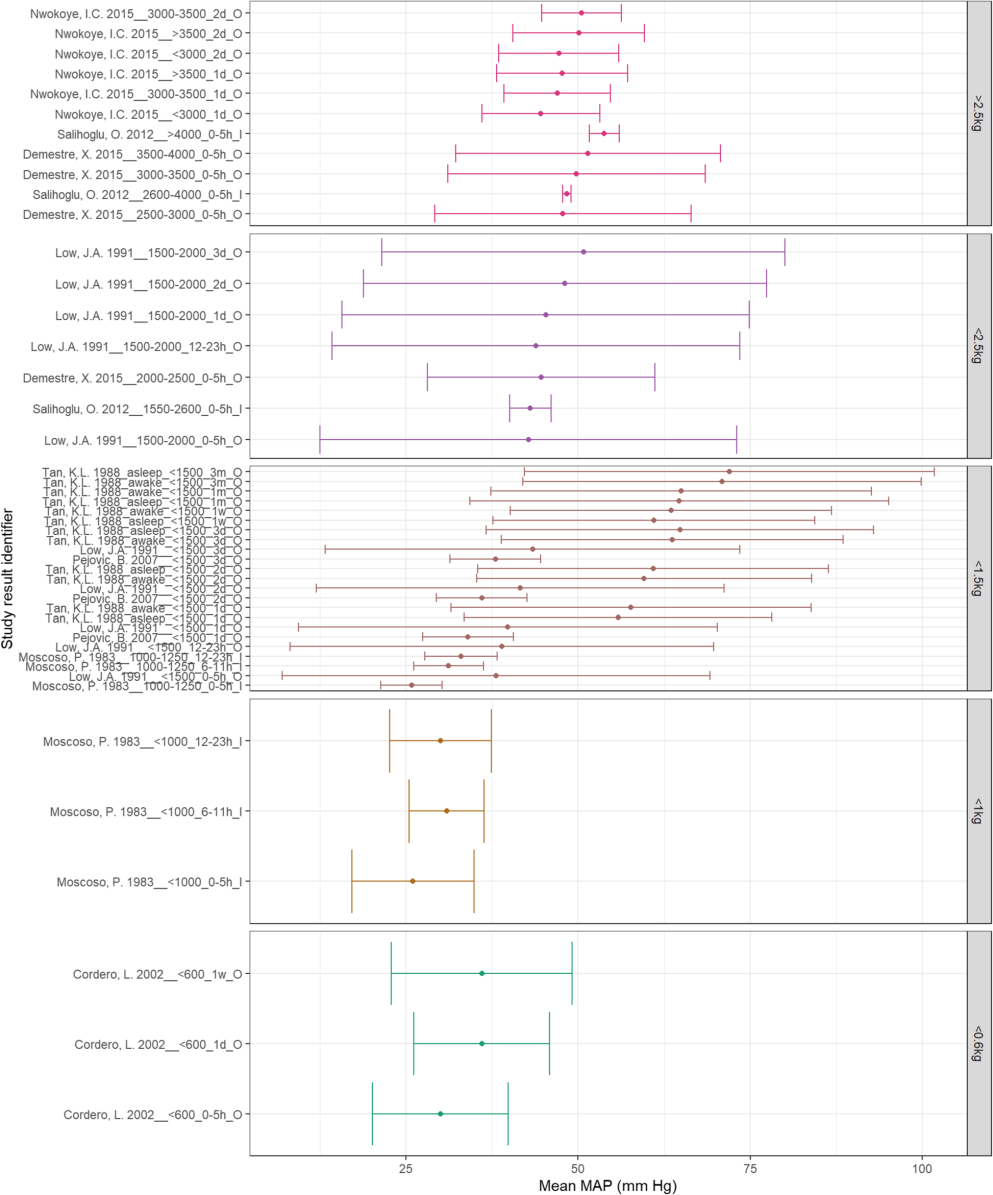
A: MAP according to BW and PNA. Mean BP (mmHg) and and 90% reference ranges (1.64SD) for neonates with normal weight (> 2.5 kg), < 2.5 kg, < 1.5 kg, < 1 kg, < 0.6 kg, including groups that span categories. Results reported from 0–5 h to 3 months of age and sorted in descending order of BW category and PNA. The labels on the vertical axis give the following information: author & year_(subgroup, if applicable)_GA_PNA_method where method is method of BP measurement used (D = Doppler, I = intra-arterial, O = oscillometric, C = non-invasive photoplethysmographic cuff, S = Sphygmomanometer)

**Fig. 12:**
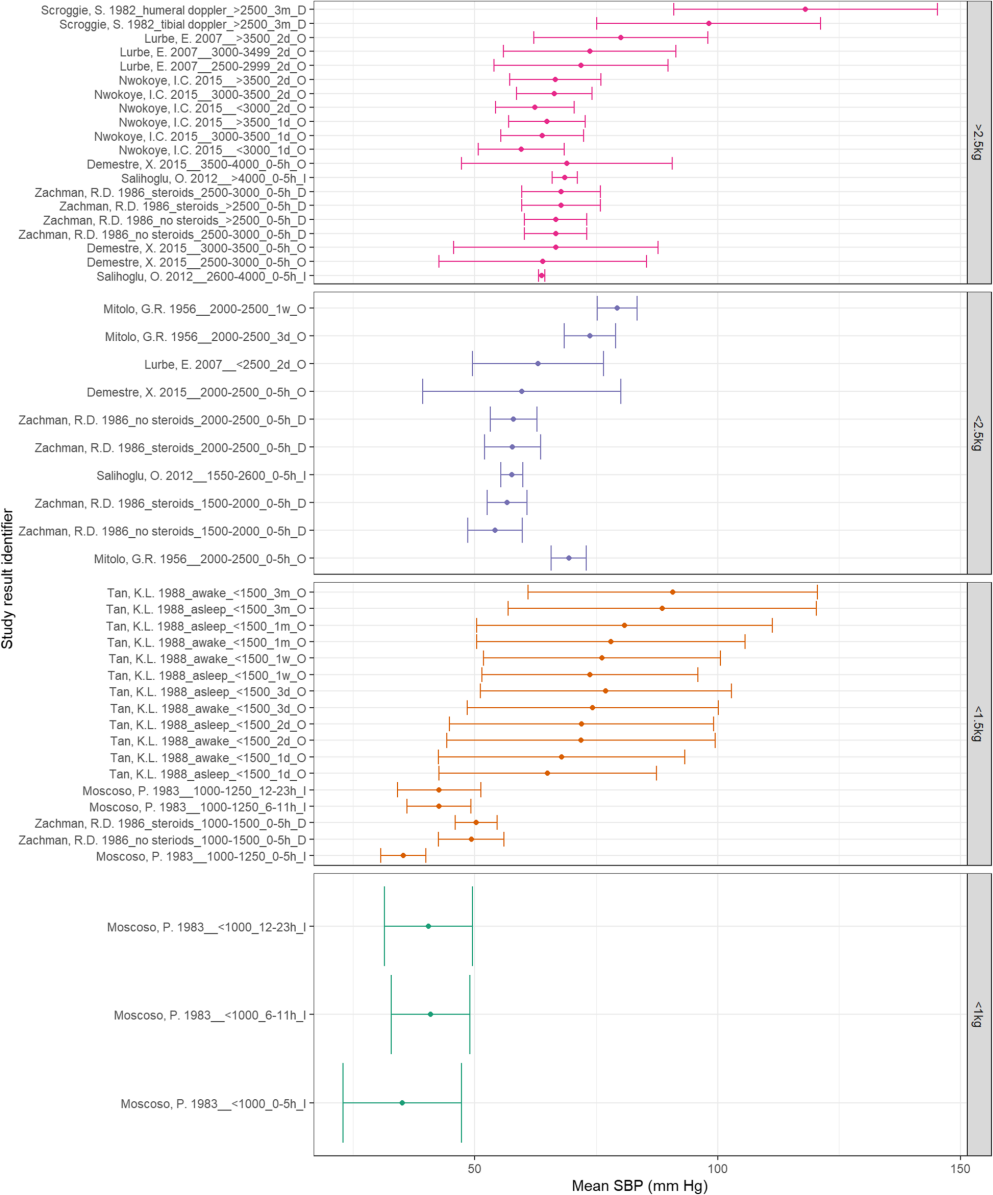
B: SBP according to BW and postnatal age. Mean BP (mmHg) and and 90% reference ranges (1.64SD) for neonates with normal weight (> 2.5 kg), < 2.5 kg, < 1.5 kg, < 1 kg, < 0.6 kg, including groups that span categories. Results reported from 0–5 h to 3 months of age and sorted in descending order of BW category and PNA. The labels on the vertical axis give the following information: author & year_(subgroup, if applicable)_GA_PNA_method where method is method of BP measurement used (D = Doppler, I = intra-arterial, O = oscillometric, C = non-invasive photoplethysmographic cuff, S = Sphygmomanometer)

**Fig. 13:**
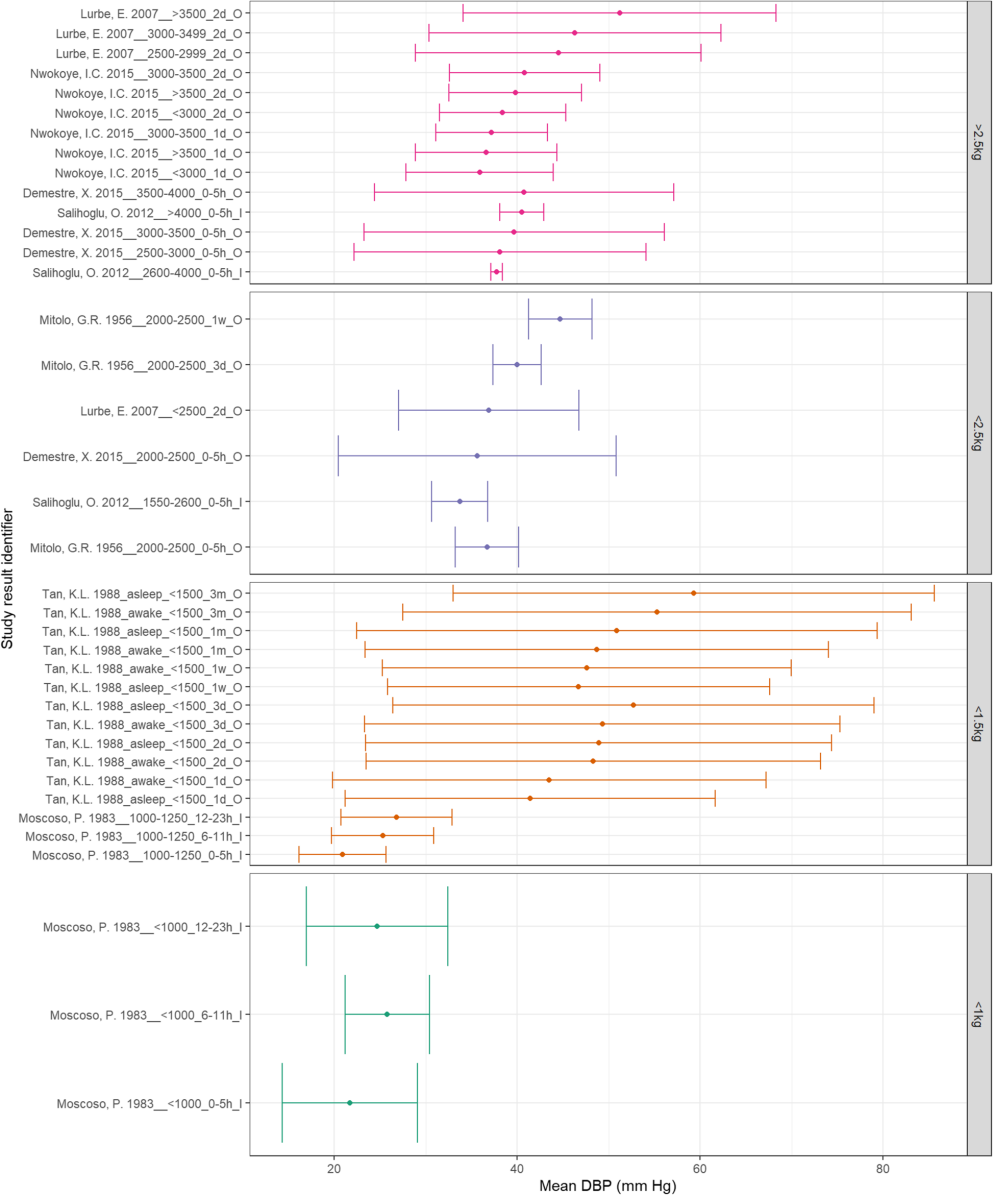
C: DBP according to BW and postnatal age. Mean BP (mmHg) and and 90% reference ranges (1.64SD) for neonates with normal weight (> 2.5 kg), < 2.5 kg, < 1.5 kg, < 1 kg, < 0.6 kg, including groups that span categories. Results reported from 0–5 h to 3 months of age and sorted in descending order of BW category and PNA. The labels on the vertical axis give the following information: author & year_(subgroup, if applicable)_GA_PNA_method where method is method of BP measurement used (D = Doppler, I = intra-arterial, O = oscillometric, C = non-invasive photople-thysmographic cuff, S = Sphygmomanometer)

Figure 11 demonstrates that MAP in lower BW categories appears to increase with increasing PNA. While MAP does appear to increase with increasing BW, the < 0.6 kg BW category demonstrate higher MAPs than the < 1 kg category. Figure 12 also does not demonstrate a clear trend between SBP and PNA in normal BW neonates within 2 days of life – but does rise by 3 months of age. All low BW categories appear to demonstrate increasing SBP with increasing PNA. Additionally, SBP appears to consistently increase with increasing BW. Finally, Fig. 13 demonstrates increasing DBP with increasing PNA for all BW categories. The normal BW neonates show a slight decline in DBP at one day PNA before rising. Additionally, DBP appears to increase consistently with increasing BW.

### Study descriptions

Overall, twelve studies included BW data with a total of 7247 neonates. Six studies included BP data for neonates with a BW > 2500 g. Demestre et al. reported oscillometric BP values in the first hour after birth in 4,850 neonates (> 2500 g) [16]. Salihoglu et al. included 937 normal weight neonates with a focus on their delivery room BPs [26]. Zachman et al. reported doppler method BP measurements in the first hour after birth for 43 neonates who were exposed to antenatal corticosteroids compared to 30 neonates who were not (> 2500 g) and found no significant difference between these groups, albeit this may have been underpowered [32]. Nwokoye et al. examined oscillometric BP values in 310 term neonates (> 2500 g) in the initial 48 h after birth and reported a statistically significant correlation between BW and SBP during the first 48 h of life (r = 0.29 $0.37 at 0–24 & 25–48 h respectively) [38]. Scroggie et al. examined BP values at 2—3 months of age and 1 -2 years of age obtained using the Doppler method in 66 neonates (2600 – 4000 g) [39]. While they reported higher BP values in the humeral versus tibial artery at 2 -3 months of age, these values were reversed with increasing PNA. Lurbe et al. reported oscillometric BP values at 2 days of postnatal age for 126 infants (> 2500 g) [36].

Six studies included BP data for low BW neonates (< 2500 g). Demestre et al. reported values for 146 neonates with a BW of 2000-2500 g and Zachman et al. reported 61 neonates without antenatal corticosteroid exposure and 59 with antenatal steroids (1500-2500 g) shortly after birth [16, 32]. Later BP values up until 3 days of life were reported by Low et al. in 15 neonates (1500-2000 g) and by Lurbe et al. in 23 neonates (< 2500 g) [35, 36]. Lurbe et al. found that preterm neonates had a lower initial BP but greater increase in BP over the first month of life with a significant association between BW and BP at birth; each additional 125 g was associated with a 1 mmHg rise in SBP. There was also some evidence of a continued effect into the first month of life. Thereafter BP remained similar between preterm and term neonates from 1—12 months of age. There were 46 neonates (< 2500 g) in the Salihoglu et al. study which identified a weak positive correlation between BW and BP (r = 0.2) [26]. Mitolo and Grassi reported oscillometric BP values over the first postnatal week for 7 neonates (2000 – 2500 g) [25].

Five studies included very low BW (< 1500 g) neonates. Zachman et al. and Moscoso et al. reported small cohorts of VLBW at 34 infants (23 with and 11 without antenatal steroids) and 10 infants respectively [32, 37]. Low et al. observed an average increase in mean BP of 7 mmHg over the first 96 h after birtßh in a cohort of 20 infants (< 1500 g). Two studies reviewing VLBW infants reported BP values beyond the first postnatal week. Pejovic et al. recorded oscillometric BP values over the first 30 days in a cohort of 373 infants, including 216 with BW < 1500 g [1]. They reported a steady increase in BP over the first month (average MBP increases of 22–51% depending on GA) that was influenced by both GA and BW. Preterm neonates showed a more rapid increase compared to term and higher BW neonates. In a cohort of 45 very preterm neonates, Tan recorded oscillometric BP values over the first 10 weeks and found that BP increased over the course of the first postnatal week, declined during the second week, then increased steadily through the remainder of the study period [40].

Two studies reported BP values for BW in neonates < 1,000 g. Cordero et al. reviewed a mixture of oscillometric and invasive BP values over the first 24 h of life for 36 “hemodynamically stable” neonates (< 600 g) compared to “unstable” neonates – only the former were included in this review [34]. Linear regression failed to demonstrate that GA or BW correlated with BP values, although this may have been underpowered. Moscoso et al. invasively monitored 9 extremely low BW neonates (< 1000 g) over the first 12 h of life. They found that these neonates experienced a notable upward trend in mean arterial BP values beginning around seven hours after birth [37].

## Discussion

Blood pressure remains one of the most commonly measured vital signs for the cardiovascular system in neonates. While BP alone is an insufficient assessment of neonatal hemodynamic status, it is an important component of cardiovascular assessment. Understanding the range of BP values observed and the potential impact of numerous factors on BP is important for ensuring the appropriate incorporation of BP values into assessment of hemodynamic stability which inform the provision of interventions for both hypotension and hypertension. This systematic review of 30 identified studies is unique from previous investigations in its comprehensive review of the available literature for systolic, diastolic, and mean arterial BP, exclusion of infants who were reported to have received BP modifying therapies, and subgrouping of neonates.

Similar to previous investigations, this review demonstrated that BP increases with increasing BW, GA, and PNA. Also consistent with previous studies, BP appears to rise more rapidly after birth for preterm and low BW infants as compared to those who are term or not low BW [1, 36]. Whilst a previous study found a decrease in BP for extremely preterm infants, this finding was not generally recapitulated in this systematic review [41]. Given the wide range in observed BP values after birth for infants of all GA, BW, and PNA ranges and the numerous factors impacting BP values, reliable “normative” data that could assist with therapeutic decision-making will likely require development of a complex algorithm involving machine learning which incorporates these and other variables. This data could feasibly be combined with additional techniques that provide information about underlying cardiac output and intravascular volume, such as echocardiogram, as well as measures of end-organ function to determine a more accurate assessment of hemodynamic status [42].

This review demonstrates the complexity in addressing the observed range of BP in these neonates, considering the presence of substantial variability within the data. Comparison between studies demonstrates this variability even when BPs are measured in neonates of a similar GA, BW, and PNA. Thus, supporting the suggestion that a “normal” BP for any given GA, BW, or PNA likely falls within a broad range and definitions based on a solitary value in a particular time period are questionable. This is further complicated by the dynamicity of BP readings within the neonate which has been shown to fluctuate with repeated readings. These can also fluctuate with the state of the neonate; are they asleep, awake, or unsettled. Additionally, different formats of BP monitoring were included such as intra-arterial, Doppler, and oscillometric methods which may result in differing readings [2]. Intra-arterial measurements are expected to be the most accurate and this was utilized for many of the extremely preterm neonates. However, oscillometric measurement was the most common method utilized within studies. Whilst this method is less accurate, it does have more ecological validity considering that for many neonates there is no clinical requirement for intra-arterial cannulation and thus the risks associated with cannulation could not be justified. Some studies have even utilized both methods and combined their results despite recognition of their incongruence [19, 34].

Multiple studies were excluded from this systematic review due to limited access to original data or summary statistics, rather than purely graphical depictions of results which often did not present data reliably. Despite this, multiple limitations remain within the broad range of studies that did meet inclusion criteria. Many of these studies have small sample sizes with opportunistic sampling methods and there is marked heterogeneity between sampling methods, time of sampling, method of sampling, and grouping of reported summary statistics. Despite there being general consensus regarding subgrouping by GA (i.e. term, late preterm, very preterm, extremely preterm) and BW (i.e. normal, low BW, very low BW, extremely low BW), these categories are not consistently represented within the studies’ data. This may partially be related to collating of groups with low numbers of neonates. Despite attempting to exclude studies that had hemodynamic instability or critical disease, it is still possible that neonates affected by PDAs, sepsis, SGA, and other underlying undetected cardiac and/or renal anomalies could have been included considering the paucity of descriptive data in most studies. Furthermore, it is not necessarily clear in multiple studies whether non-arterial BP measures were pre- or post-ductal despite inclusion of neonates with a patent ductus arteriosus but without hemodynamic instability. The degree of respiratory support is another area which has found to have an effect on BP, but which was typically not comprehensively reported within the literature. In addition to this, biases pertaining to BP measurement such as neonatal state, cuff size, limb use, assessment frequency, and assessor status were typically not reported and could indeed have a contributory effect on BP measurements [2]. Notably, these parameters may variably affect neonates that fall into different prematurity or weight subgroups.

While the provision of meta-analysed results was part of the a priori research plan, this was not considered possible nor appropriate considering the degree of variation present within the studies. Therefore, a quantitative bias assessment was also not undertaken. Studies typically did not include power/sample size calculations to support claims associated with any statistical testing. Therefore, it was not surprising when a study appeared to be underpowered to find a significant contribution of BW or GA to BP [34]. In view of this, future presentation of summary statistics that use universally accepted subgroups and standard postnatal timepoints for measurement would ensure that appropriate statistical analyses could be implemented more effectively. Finally, whilst this review has been undertaken as part of an international consortium, it is worth noting that there have been further studies evaluating neonatal blood pressure since this review which demonstrate agreement with our overall findings [43, 44].

Additionally, while this review’s criteria ensured the exclusion of neonates clinically deemed to be hemodynamically instable, it is worth noting that difficulty in defining hypotension may have resulted in inappropriate exclusions being applied. Similarly, the concept of neonatal hypertension has been recognized since the 1970s, yet its identification has been complicated by the lack of effective nomograms, despite the NICU population being at increased risk [45]. Adequate identification of the upper limits of normality remains challenging and current advice recommends the use of standardized percentile cut-offs (i.e., considering treatment when consistently above the 99th percentile). Between one quarter and one half of identified hypertension in neonates using this method is not treated [46, 47]. This may be related to uncertainty regarding the expected BP values or possible treatments available to neonates. Ideally, the provision of representative nomograms will guide therapeutic interventions, investigation of underlying etiology, and appropriate short and long-term monitoring [7]. Furthermore, it has been recognized that the commonest etiology for hypertension seems to differ between preterm neonates where perinatal factors are typically implicated, and term neonates where underlying systemic diseases appear to be most contributory [48]. This variable etiology demonstrates the importance of distinguishing between these sub-groups to determine not only the prevalence of hypertension, but also to understand any differences in both acute and chronic outcomes.

## Conclusions

Overall, this review demonstrates that BW and GA are both positively correlated with higher BP values which continue to increase throughout the postnatal period. We have shown that there is high variation both within and between studies that have attempted to assess the observed range of BP values in neonates – demonstrating the dynamic nature of BP and the variability within populations. Future collaborative research should develop a standard approach to subgrouping of BW/GA and an appropriate statistical plan and analysis of multi-centre patient data to provide reliable, collatable, and thus informative observed ranges.

### Supplementary Information

**Additional file 1. **Detailed Methods.

**Additional file 2. **PRISMA 2020 Checklist.

**Additional file 3. **All studies included in the systematic review.

**Additional file 4. **Extracted study data.

## Data Availability

No datasets were generated or analysed during the current study.

## Abbreviations

BP: Blood pressure(s)
BW: Birth weight
DBP: Diastolic BP
GA: Gestational age
MAP: Mean arterial pressure
NICU: Neonatal intensive care unit(s)
PNA: Postnatal age
SD: Standard deviation
SBP: Systolic BP
mmHg: Millimeters of mercury
