# Supplementary material for: Neonatal blood pressure by birth weight, gestational age, and postnatal age: a systematic review
Source: Matern Health Neonatol Perinatol. 2024 May 1;10:9. doi: 10.1186/s40748-024-00180-w (PMC11061963; doi:10.1186/s40748-024-00180-w)
Supplement: Supplementary file 1 — Additional file 1. Detailed Methods. [file 40748_2024_180_MOESM1_ESM.docx]

**Detailed Methods**

A literature search was conducted by a professional librarian in MEDLINE, PubMed, Embase, the Cochrane Library, and CINAHL. The search terms were (blood pressure OR hypertension OR hypotension) AND (infant OR newborn OR neonate) AND infant [MeSH] AND (measurement OR normative) AND humans [MeSH]. Inclusion criteria included prospective or retrospective cohorts, case series, and randomized controlled trials. Exclusion criteria included absence of extractable data in letters, comments, papers, and reviews. The search included publications from January 1946 to December 2017 and did not limit year of publication because some of the earliest studies provide evidence for intra-arterial and oscillometric BP measurements. Unpublished data from research undertaken by consortium members was also considered for inclusion. The study population included neonates from birth, with expansion to infants up to 3 months PNA where data were available. Studies that included neonates with major congenital anomalies (e.g., cardiac defects) were excluded from consideration. Studies involving hemodynamically compromised and critically unwell neonates requiring blood-pressure modifying agents such as volume expansion or inotropes were excluded. Studies were also excluded if they did not contain either individualized patient data and/or descriptive summary statistics with information about data spread. Furthermore, studies that presented SGA (small for gestational age) data without BW and/or GA were not included. Although 70 non-English-language articles were identified, all were reviewed except for 13 in languages including Bulgarian, Czech, Hungarian, and Russian because they were unable to be translated by workgroup members. All co-authors participated in study review and data extraction. The first stage of data extraction included bibliographic details, study population including GA and PNA, study design, any therapeutic interventions, method of BP measurement, outcome measures, description of results, and study limitations. Study results were then extracted for in-depth review following assessment indicating a probable link to the review questions. This stage involved a more detailed consideration of the descriptive statistics presented in the selected articles by at least two independent reviewers with extraction of BP measurements over time as grouped by BW and/or GA, sample size and setting, and any relevant demographic differences within study populations. Study bias and certainty was qualitatively assessed by at least two independent reviewers and assigned as low, medium or high with explanatory comments. All relevant results were extracted by two independent reviewers regardless of sample size and format of BW and/or GA subgroups. Notably, data presented only within graphs without explicit numerical values were not extracted. Reporting of methods and results are in accordance with the PRISMA 2020 Standard (Supplementary Table 1).^1^ The protocol for this review is registered on the PROSPERO website with ID CRD42018092886.

Planned analysis of extracted data was descriptive as formal statistical comparisons were not possible. Types of data extracted from studies for systolic blood pressure (SBP), diastolic blood pressure (DBP) and mean arterial pressure (MAP) included: minimum to maximum values, mean, standard deviation (SD), interquartile range (i.e. 25^th^ to 75^th^ percentile), 10^th^ to 90^th^ percentile, reference or normal range (i.e. mean ± 1.96×SD or 2×SD), median and interquartile range were extracted if available. Samples sizes were also collected. Data were extracted and collated into the subgroups for BW and GA as suggested by Ward et al^2^. The <600g BW category was added to provide consistency with the lowest BW grouping from studies identified within the literature search.

BW categories were defined as:

1. Low BW (LBW) infants: <2,500 g BW

2. Very low BW (VLBW) infants: <1,500 g BW

3. Extremely low BW infants (ELBW) infants: <1,000 g BW

4. Preterm infants with a BW <600 g

GA (at birth) categories were classified as follows:

1. Post term neonates: ≥ 42^0/7^ weeks GA
2. Term neonates: 37^0/7^ to 41^6/7^ weeks GA
3. Moderate to late preterm neonates: 32^0/7^ to 36^6/7^ weeks GA
4. Very preterm neonates: 28^0/7^ to 31^6/7^ weeks GA
5. Extremely preterm infant: ≤ 27^6/7^ weeks GA

Ninety percent (90%) reference ranges were calculated as mean±1.64×SD. For studies that reported standard errors or confidence intervals instead of standard deviations (SDs), SDs were calculated from the quantities stated using standard formulae.

1. Page MJ, McKenzie J, Bossuyt P, Boutron I, Hoffmann T, Mulrow C, et al. The PRISMA 2020 statement: An updated guideline for reporting systematic reviews. PLoS Med*.* 2021;18.
2. Ward RM, Benjamin D, Barret J, Allagaert K, Portman R, Davis J, et al. Safety, dosing, and pharmaceutical quality for studies that evaluate medicinal products (including biological products) in neonates. Pediatr Res*.* 2017;81:692–711.
