# Supplementary material for: Neonatal blood pressure by birth weight, gestational age, and postnatal age: a systematic review
Source: Matern Health Neonatol Perinatol. 2024 May 1;10:9. doi: 10.1186/s40748-024-00180-w (PMC11061963; doi:10.1186/s40748-024-00180-w)
Supplement: Supplementary file 3 — Additional file 3. All studies included in the systematic review. [file 40748_2024_180_MOESM3_ESM.docx]

**Table 1.** All studies included in the systematic review.

| **Authors** | **Year** | **Title** | **Journal and Citation** | **Country** | **Recruited** | **Design** | **BP Measurement Method** | **GA Data** | **BW Data** | **Bias Risk** | **Comments** |
| --- | --- | --- | --- | --- | --- | --- | --- | --- | --- | --- | --- |
| Batton B, et al. | 2007 | Blood pressure during the first 7 days in premature infants born at postmenstrual age 23 to 25 weeks. | Am J Perinatol. 2007;24:107–116 | United States | 142 | Retrospective cohort | Intra-arterial (umbilical) or oscillometric | X |  | Low |  |
| Batton, B., et al. | 2014 | Evolving blood pressure dynamics for extremely preterm infants. | Journal of Perinatology, 2014. 34(4): p. 301-305. | United States | 367 | Prospecitve cohort | Intra-arterial (umbilical) or cuff | X |  | Moderate | Selection (inborns only), intervariability across NICUs, treatment was at providers' discretion |
| Cordero L, et al. | 2002 | Mean arterial pressures during the first 24 hours of life in < or = 600-gram birth weight infants. | Journal of perinatology : official journal of the California Perinatal Association, 2002. 22(5): p. 348-353. | United States | 36 | Retrospective cohort | Oscillometric for admission BP, then intra-arterial (umbilical) |  | X | Low | Small sample size |
| de Swiet M, et al. | 1992 | Blood pressure in the first 10 years of life: the Brompton study | British Medical Journal 1992; 304:23-26 | United Kingdom | 1895 | Prospective cohort | Doppler | X |  | Low |  |
| de Swiet M, et al. | 1980 | Systolic blood pressure in a population of infants in the first year of life: the Brompton study. | Pediatrics 1980;65:1028-1035 | United Kingdom | 1895 | Prospective cohort | Doppler | X |  | Low | Possible detection bias |
| Demestre X, et al. | 2015 | Blood pressure values in healthy newborns. | Pediatria Catalana, 2015. 75(3): p. 107-111. | Spain | 4996 | Prospective cohort | Ocillometric | X | X | Low |  |
| Earley A, et al. | 1980 | Blood pressure in the first 6 weeks of life. | Archives of disease in childhood, 1980. 55(10): p. 755-757. | UK | 99 | Prospective cohort | Non-invassive cuff | X |  | Low |  |
| Gemelli M, et al. | 1990 | Longitudinal study of blood pressure during the 1st year of life | European Journal of Pediatrics 1990; 149:318-320 | Italy | 514 | Prospective cohort | Oscillometric | X |  | Low |  |
| Iwami H, et al | Data collection 2015-2016. Published 2019, received formal subset data 2020. | Unpublished data from PLASE Study which is described in: Toyoshima et al. What echocardiographic indices are predictive of patent ductus arteriosus surgical closure in early preterm infants? A prospective multicenter cohort study. J Cardiol  . 2019 Dec;74(6):512-518. | Unpublished data. | Japan | 710 | Prospective cohort | Intra-arterial and oscillometric | X |  | Uncertain |  |
| Kang C, et al. | 2016 | Dynamic Changes of Pulmonary Arterial Pressure and Ductus Arteriosus in Human Newborns From Birth to 72 Hours of Age. | Medicine, 2016. 95(3): p. e2599. | China | 76 | Prospective cohort | Oscillometric | X |  | Low | Small sample size |
| Low JA, et al. | 1991 | Blood pressure and heart rate of the preterm newborn following delivery. Clinical and investigative medicine. | Medecine clinique et experimentale, 1991. 14(3): p. 183-187. | Canada | 35 | Prospective cohort | Oscillometric |  | X | Low | Grouped data may under or over estimate norms, small sample size |
| Lurbe E, et al. | 2007 | First-year blood pressure increase steepest in low birthweight newborns. | Journal of hypertension, 2007. 25(1): p. 81-86. | Spain | 464 | Prospective cohort | Oscillometric |  | X | Uncertain | Attrition bias |
| Menghetti E, et al. | 1988 | Dynamic monitoring of neonatal blood pressure using the oscillometric method. Monitoraggio dinamico della pressione neonatale con metodica oscillometrica. | La Pediatria medica e chirurgica (medical and surgical pediatrics), 1988. 10(2): p. 169-175. | Italy | 160 | Prospective cohort | Oscillometric | X |  | Low | Selection bias |
| Menghetti E, et al. | 1993 | Monitoring for 15 minutes the arterial pressure in 201 healthy neonates with or without familiarity for hypertension. | European Review for Medical and Pharmacological Sciences, 1993. 15(3-4): p. 159-162. | Italy | 201 | Prospective cohort | Oscillometric | X |  | Low |  |
| Menghetti E, et al. | 1995 | Variability in the arterial pressure of newborns as compared to that of adults and old men. | La Clinica terapeutica, 1995. 146(10): p. 617-621. | Italy | 108 | Prospective cohort | ABPM | X |  | Low |  |
| Menghetti E, et al. | 1995 | Early detection by non-invasive monitoring of abnormally elevated systolic blood pressure in newborns with a positive familiarity for hypertension. | Recenti progressi in medicina, 1995. 86(5): p. 195-197. | Italy | 263 | Prospective cohort | Oscillometric | X |  | Low |  |
| Mitolo, et al. | 1956 | Remarks on arterial BP in the neonate | Pathologica, Vol XLVIII, 1956 | Italy | 95 | Prospective cohort | Oscillometric | X | X | Low |  |
| Moscoso P, et al. | 1983 | Spontaneous elevation in arterial blood pressure during the first hours of life in the very-low-birth-weight infant. | Journal of Pediatrics, 1983. 103(1): p. 114-117. | United States | 25 | Prospective cohort | Intra-arterial (umbilical) |  | X | Low | Small sample size |
| Nwokoye IC, et al. | 2015 | Blood pressure values in healthy term newborns at a tertiary health facility in Enugu, Nigeria | Nigerian Journal of Clinical Practice, 2015;18(5)584 | Nigeria | 310 | Prospective cohort | Oscillometric |  | X | Low |  |
| Pejovic B, et al. | 2007 | Blood pressure in non-critically ill preterm and full-term neonates. | Pediatric nephrology (Berlin, Germany), 2007. 22(2): p. 249-257. | Serbia | 373 | Prospective cohort | Oscillometric | X | X | High | Measurement bias: concerns regarding the type of oscillometric device |
| Salihoglu O, et al. | 2012 | Delivery room blood pressure percentiles of healthy, singleton, liveborn neonates. | Pediatrics International, 2012. 54(2): p. 182-189. | Turkey | 982 | Cross-sectional | Intra-arterial | X | X | Low |  |
| Samanta M, et al. | 2015 | Normative blood pressure data for Indian neonates. | Indian pediatrics, 2015. 52(8): p. 669-673. | India | 1617 | Prospective cohort | Oscillometric | X |  | Low |  |
| Samanta M. et al. | 2015 | Blood pressure variation with gestational age and birth weight in indian newborn. | Journal of Tropical Pediatrics, 2015. 61(3): p. 197-205. | India | 1617 | Prospective cohort | Oscillometric | X |  | Low |  |
| Satoh M, et al. | 2016 | Reference values and associated factors for Japanese newborns' blood pressure and pulse rate: The babies' and their parents' longitudinal observation in Suzuki Memorial Hospital on intrauterine period (BOSHI) study. | Journal of Hypertension, 2016. 34(8): p. 1578-1585. | Japan | 2628 | Cross-sectional | Oscillometric | X |  | Low |  |
| Schachter J, et al. | 1982 | Blood pressure during the first two years of life. | American journal of epidemiology, 1982. 116(1): p. 29-41. | United States | 392 | Prospective cohort | Intra-arterial and ultrasonic | X |  | Low |  |
| Scroggie S, et al. | 1982 | Indirect simultaneous measurement of arterial blood pressure in infants by 3 methods. Medicion simultanea indirecta de la presion arterial en lactantes mediante tres metodos. | , 1982. 53(6): p. 558-561. | Chile | 212 | Prospective cohort | Doppler, flush, and auscultation |  | X | Low |  |
| Tan KL, et al. | 1988 | Blood pressure in very low birth weight infants in the first 70 days of life. | Journal of Pediatrics, 1988. 112(2): p. 266-270. | Singapore | 70 | Prospective cohort | Oscillometric |  | X | Low | Small sample size |
| Vilarim, JN, et al. | 2000 | Systolic and diastolic blood pressure levels of healthy newborn infants. Niveis de pressao arterial sistolica e diastolica. | J Pediatr (Rio J), 2000. 76(4): p. 287-289. | Brazil | 641 | Prospective cohort | Oscillometric | X |  | Unclear |  |
| Witcombe NB, et al. | 2008 | Blood pressure and heart rate patterns during sleep are altered in preterm-born infants: Implications for sudden infant death syndrome. | Pediatrics, 2008. 122(6): p. e1242-e1248. | Australia | 45 | Prospective cohort | Oscillometric | X |  | Unclear | Small sample size, sample method not described |
| Zachman RD, et al. | 1986 | Neonatal blood pressure at birth by the Doppler method | Am Heart J 1986; 111:189. | United States | 225 | Cross-sectional | Doppler | X | X | Low | Sample bias, possibility of slight measurement bias |
